# An African-specific haplotype in *MRGPRX4* is associated with menthol cigarette smoking

**DOI:** 10.1371/journal.pgen.1007916

**Published:** 2019-02-15

**Authors:** Julia Kozlitina, Davide Risso, Katherine Lansu, Reid Hans Johnson Olsen, Eduardo Sainz, Donata Luiselli, Arnab Barik, Carlos Frigerio-Domingues, Luca Pagani, Stephen Wooding, Thomas Kirchner, Ray Niaura, Bryan Roth, Dennis Drayna

**Affiliations:** 1 McDermott Center for Human Growth and Development, University of Texas Southwestern Medical Center, Dallas, Texas, United States of America; 2 National Institute on Deafness and Other Communication Disorders, National Institutes of Health, Bethesda, Maryland, United States of America; 3 Division of Chemical Biology and Medicinal Chemistry, Eshelman School of Pharmacy, University of North Carolina School of Medicine, Chapel Hill, North Carolina, United States of America; 4 Department of Biological, Geological, and Environmental Sciences, University of Bologna, Bologna, Italy; 5 National Center for Complementary and Integrative Health, National Institutes of Health, Bethesda, Maryland, United States of America; 6 Estonian Biocentre, Institute of Genomics, University of Tartu, Estonia; 7 School of Public Health, University of California, Merced, California, United States of America; 8 Schroeder Institute for Tobacco Research, Washington, District of Columbia, United States of America; Stanford University School of Medicine, UNITED STATES

## Abstract

In the U.S., more than 80% of African-American smokers use mentholated cigarettes, compared to less than 30% of Caucasian smokers. The reasons for these differences are not well understood. To determine if genetic variation contributes to mentholated cigarette smoking, we performed an exome-wide association analysis in a multiethnic population-based sample from Dallas, TX (N = 561). Findings were replicated in an independent cohort of African Americans from Washington, DC (N = 741). We identified a haplotype of *MRGPRX4* (composed of rs7102322[G], encoding N245S, and rs61733596[G], T43T), that was associated with a 5-to-8 fold increase in the odds of menthol cigarette smoking. The variants are present solely in persons of African ancestry. Functional studies indicated that the variant G protein-coupled receptor encoded by *MRGPRX4* displays reduced agonism in both arrestin-based and G protein-based assays, and alteration of agonism by menthol. These data indicate that genetic variation in *MRGPRX4* contributes to inter-individual and inter-ethnic differences in the preference for mentholated cigarettes, and that the existence of genetic factors predisposing vulnerable populations to mentholated cigarette smoking can inform tobacco control and public health policies.

## Author summary

An exome-wide association study revealed a significant association between menthol cigarette use and coding variants in *MRGPRX4*, which encodes a G-protein coupled receptor expressed in sensory neurons. The variant haplotype is found only in populations of African ancestry, and encodes a receptor that displays reduced agonism by Nateglinide. Our findings indicate genetic variation contributes to the high rate of menthol cigarette use in African Americans.

## Introduction

Cigarette smoking remains a leading cause of preventable disease and mortality in the United States, contributing to >480,000 deaths annually [1]. Although the overall rates of smoking have declined dramatically over the last 50 years [1], the use of mentholated cigarettes has not, and has actually increased in some groups [2, 3]. Menthol is a flavoring additive commonly used in cigarettes and tobacco products. It is thought to reduce the harshness of cigarette smoke due to its cooling and anesthetic properties [4–6]. Menthol cigarettes currently account for about 30% of the cigarette market in the U.S. [7]. Scientific evidence suggests that the use of mentholated cigarettes leads to increased smoking initiation among youth and reduced rates of cessation [8, 9]. This has led the FDA to conclude that menthol cigarettes likely pose a public health risk above that of nonmenthol cigarettes [10, 11].

The prevalence of menthol cigarette smoking varies markedly between demographic groups, and is especially high among young adults and in African Americans [3, 9, 12]. In the U.S., nearly 83% of African-American smokers use menthol cigarettes, compared to 24% of white and 32% of Hispanic smokers. Whether this disparity has a genetic basis, or is attributable solely to social or cultural factors, is not known.

Menthol is known to interact with transient receptor potential (TRP) channels, including TRPM8 [13] and TRPA1 [14]. Although one study found that common variants in *TRPA1* were associated with menthol tobacco use among European-American smokers [15], this finding awaits replication. Variations in the *TAS2R38* bitter taste receptor gene appear to have a modest effect on smoking and on menthol cigarette use [16–20], but no comprehensive analysis of the role of variation in these and other genes in menthol cigarette smoking has been carried out to date.

To determine whether inherited variations in the protein-coding regions of the genome contribute to menthol cigarette smoking, we performed an exome-wide association study using a population-based cohort of African Americans (AA) and European Americans (EA) from Dallas, Texas. The findings were replicated in a cohort of African-American smokers from Washington, DC.

## Results

### Study cohort

The discovery cohort included 561 participants (394 AA and 167 EA) from the Dallas Heart Study (DHS) and the Dallas Biobank (Table 1). The average age of participants was 55±11.0 (SD) years, and 60% were women. Nearly 78% of DHS AA and 86% of Biobank AA subjects reported smoking mentholated cigarettes, compared to 33% of European Americans (P<0.001), consistent with national trends [3]. Menthol smokers were younger than non-menthol smokers among African Americans (P<0.05), but there was no difference in age among European-American smokers. The prevalence of menthol smoking was not significantly different between DHS and Biobank AA after adjusting for age (P = 0.59). In the replication cohort (Schroeder), most of the participants (N = 424, 57.2%) were menthol smokers (Table 1). A higher percentage of menthol smokers than non-menthol smokers were female (39.6% versus 24.3%, P<0.001) consistent with previous literature [12]. No differences were found in the mean age of menthol smokers and non-menthol smokers (P = 0.41).

**Table 1:** Characteristics of study participants according to menthol smoking status.

Population	Characteristic	Non-Menthol Smokers	Menthol Smokers	P-value
DHS AA (N = 261)	N	58 (22.2)	203 (77.8)	-
	Age (yr)	62.8 ± 9	56.4 ± 9.1	<0.0001
	Female, no. (%)	34 (58.6)	124 (61.1)	0.762
Biobank AA (N = 133)	N	19 (14.3)	114 (85.7)	-
	Age (yr)	56.4 ± 11.8	47.2 ± 12.5	0.0031
	Female, no. (%)	12 (63.2)	72 (63.2)	1
DHS EA (N = 167)	N	112 (67.1)	55 (32.9)	**-**
	Age (yr)	55.9 ± 9.3	55.3 ± 10.3	0.75
	Female, no. (%)	59 (52.7)	33 (60.0)	0.4105
Schroeder AA (N = 741)	N	317 (42.8)	424 (57.2)	-
	Age (yr)	44.6 ± 10.6	45.3 ± 10.9	0.41
	Female, no. (%)	77 (24.3)	168 (39.6)	<0.0001

Values are mean ± SD. AA–African American; EA–European American.

P-values were calculated using t-tests (for age) and chi-square tests (for gender).

### Identification of *MRGPRX4*

A total of 52,298 variants were tested for association with menthol cigarette smoking in the Dallas cohort. Genomic control [21] value was acceptable (λ_gc_ = 1.05) and QQ-plot of P-values showed no systematic inflation of association results (S1 Fig). No variant met our exome-wide significance threshold (9.6x10^-7^). We therefore decided to investigate the top variants with a suggestive level of significance (P<1x10^-4^) in greater detail. A total of three variants reached this level of significance in our exome-wide screen (Table 2), and these were genotyped in an additional cohort of 741 AA smokers from Washington DC (Schroeder cohort). While no association was found with two of the three variants (Table 2), the third variant, rs7102322 in the gene *MRGPRX4*, was strongly associated with menthol smoking in the replication cohort (P = 2.1x10^-6^). Meta-analysis of the two samples together revealed an even lower P-value that exceeded criteria for genome-wide significance (P = 1.6x10^-8^, Table 2).

**Table 2:** Top association results from exome-wide analysis.

					Discovery cohort (N = 561)			Replication cohort (N = 741)			Meta-analysis P-value
Chr	GRCh37/hg19 Position	rs ID	Gene	Ref/Alt	Alt AF	OR (95% CI)	P-value	Alt AF	OR (95% CI)	P-value	
17	38156712	rs4794822	*PSMD3*	G/A	0.34	0.53 (0.39–0.72)	4.6E-05	0.33	1.28 (0.99–1.59)	0.06	0.052
11	18195537	rs7102322	*MRGPRX4*	A/G	0.06	8.55 (2.04–35.9)	4.8E-05	0.05	6.3 (2.90–13.4)	2.1E-06	1.6E-08
3	142178144	rs2229032	*ATR*	G/A	0.10	2.81 (1.63–4.86)	8.6E-05	0.08	0.96 (0.65–1.43)	0.83	0.37

Ref–reference allele, Alt–alternate allele, AF–allele frequency, OR–odds ratio. In the Discovery cohort, the P-values were calculated using logistic regression adjusted for age, gender, and 6 leading principal components of ancestry (based on a likelihood-ratio test). In the replication cohort, P-values were calculated using logistic regression adjusted for age and gender. The combined p-values were calculated using random-effects inverse-variance weighted meta-analysis. Odds ratios were calculated assuming additive genetic model.

The rs7102322 variant was seen exclusively in African-American participants (minor allele frequency [MAF] = 8% in the Dallas cohorts, 5% in Schroeder) and was not observed in European Americans (0% in DHS EA). Among the AA participants in the Dallas cohorts, the allele frequency of the variant was five-to-eight fold higher in menthol smokers compared to non-menthol smokers (10.4% vs 1.3%, odds ratio (OR) = 8.5, P = 5.6x10^-5^ (Table 3). A similar magnitude of difference was seen in the Schroeder cohort (7.0% vs 1.3%, OR = 6.3, P = 2.1x10^-6^, Table 3). Although limited by low power, our analyses found highly similar differences in the *MRGPRX4* allele frequencies between menthol and non-menthol smokers in males and females (S1 Table). To determine whether the lower frequency of the rs7102322 variant in the Schroeder cohort (5%) was influenced by admixture, we estimated the percentage of African and European ancestry in a subset of this cohort. This indicated that Schroeder cohort participants indeed have a higher degree of European admixture compared to West Africans and African Americans from other regions of the U.S. (S2 Fig). Further analyses that included ancestry informative markers and an inferred proportion of African ancestry at this locus maintained strong support for association with menthol smoking (P<1e-5), indicating that the observed association is unlikely to be due to differential admixture (S4 and S5 Tables).

**Table 3:** Association of *MRGPRX4* rs7102322 with menthol smoking in African-American participants.

		rs7102322 genotype				
Population	Smoking status	A/A	A/G	G/G	MAF	P-value
DHS	Non-menthol	57	1	0	0.9%	
	Menthol	165	34	4	10.3%	0.00014
	% Menthol	74.3%	97.1%	100.0%		
Biobank	Non-menthol	18	1	0	2.6%	
	Menthol	90	24	0	10.5%	0.079
	% Menthol	83.3%	96.0%	-		
DHS + Biobank	Non-menthol	75	2	0	1.3%	
	Menthol	255	58	4	10.4%	5.65E-05
	% Menthol	77.3%	96.7%	100.0%		
Schroeder	Non-menthol	309	8	0	1.3%	
	Menthol	365	59	0	7.0%	2.06E-06
	% Menthol	54.1%	88.1%	-		

MAF—minor allele frequency

P-values were calculated using logistic regression adjusted for age and gender. In addition, cohort indicator was included as a covariate in the combined DHS + Biobank analysis.

### Characterization of *MRGPRX4* variation and function

**Fig 1:**
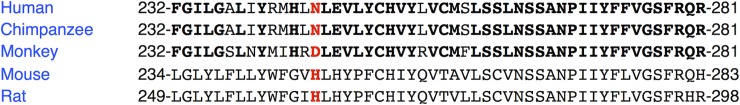
*MRGPRX4* multiple sequence alignment. Bolded letters denote residues that are conserved among primates. Residue 245 is shown in red.

The *MRGPRX4* gene encodes a Mas-related G-protein coupled receptor member X4, which is expressed in nociceptive neurons of the dorsal root ganglia and trigeminal neurons, and may regulate pain and somatosensation [22–24]. The *MRGPRX4* rs7102322 variant encodes an asparagine-to-serine substitution at codon 245 (N245S). The residue is conserved in chimpanzees, and resides immediately 5’ to a region highly conserved across primates (Fig 1).

To evaluate whether rs7102322 SNP was in linkage disequilibrium (LD) with another functional variant in the *MRGPRX4* locus, we examined data from the 1000 Genomes Project [25]. Consistent with our observations, the rs7102322 variant was observed solely in African-ancestry populations (MAF = 11.5% in Africans and 8% in African Americans in Southwest U.S.). The rs7102322 variant was in LD with the SNP rs61733596[A/G], which encodes a synonymous substitution at codon 43 (T43T) in *MRGPRX4*. Genotyping the rs61733596 variant in the Schroeder cohort confirmed that this variant is in complete linkage disequilibrium (R^2^ = 1) with rs7102322.

### Sequencing of *MRGPRX4*

To identify whether additional coding variants in *MRPGRX4* were associated with menthol cigarette smoking, we sequenced all exons of *MRPGRX4* in a subset of Dallas cohort participants (N = 389, Table 4). This analysis confirmed that the rs7102322 (N245S) variant was in complete LD with rs61733596 (T43T), which showed an equivalent association with menthol smoking (OR = 3.3, P = 0.007). No other coding variant in *MRGPRX4* was in linkage disequilibrium with N245S or associated with menthol cigarette smoking in this group.

**Table 4:** *MRGPRX4* variants identified by exome sequencing in Dallas cohort participants (N = 389).

CHR	GRCh37/hg19 Position	SNP	Ref/Alt	MAF Menthol (N = 227)	MAF Non-menthol (N = 162)	OR (95% CI)	P-value	Variant Effect
11	18194793	rs11024530	G/A	13.9%	14.8%	1.36 (0.86–2.13)	0.1801	5' UTR
11	18194827	rs2468774	C/G	26.4%	26.2%	1.06 (0.76–1.49)	0.7227	F8L
11	18194878	rs2445180	T/G	26.4%	25.6%	1.09 (0.78–1.53)	0.6125	N25K
11	18194932	rs61733596	A/G	6.6%	1.5%	3.34 (1.25–8.89)	**0.0067**	T43
11	18194944	rs11024531	T/A	14.8%	15.1%	1.39 (0.89–2.17)	0.1440	V47
11	18194964	rs1869788	A/G	37.7%	32.7%	1.16 (0.84–1.58)	0.3622	Y54C
11	18194996	rs144828790	A/C	0.7%	0.9%	0.52 (0.13–2.13)	0.3710	I65L
11	18195051	rs2445179	C/T	8.6%	6.2%	0.97 (0.54–1.73)	0.9204	L83S
11	18195173	rs61733597	G/A	0.9%	1.2%	0.56 (0.13–2.34)	0.4299	V124I
11	18195227	rs78287429	G/A	9.0%	8.6%	0.71 (0.41–1.22)	0.2181	V142M
11	18195252	rs73434269	C/T	2.2%	1.9%	0.81 (0.30–2.18)	0.6774	S150F
11	18195347	rs113302139	G/T	0.9%	0.3%	1.98 (0.22–17.98)	0.5195	A182S
11	18195348	rs11024532	C/T	10.8%	17.6%	0.83 (0.54–1.27)	0.3925	A182V
11	18195448	rs4630269	C/T	26.2%	25.9%	1.07 (0.76–1.50)	0.7039	Y215
11	18195537	rs7102322	A/G	6.6%	1.5%	3.34 (1.25–8.89)	**0.0067**	N245S
11	18195609	rs146132319	C/T	0.4%	0.6%	1.36 (0.18–10.23)	0.7661	P269L

P-values were calculated using logistic regression adjusted for age, gender, and self-reported ancestry (African American vs European American). Bolded numbers indicate P<0.05.

### MRGPRX4 N245S mutation attenuates agonist activity

**Fig 2:**
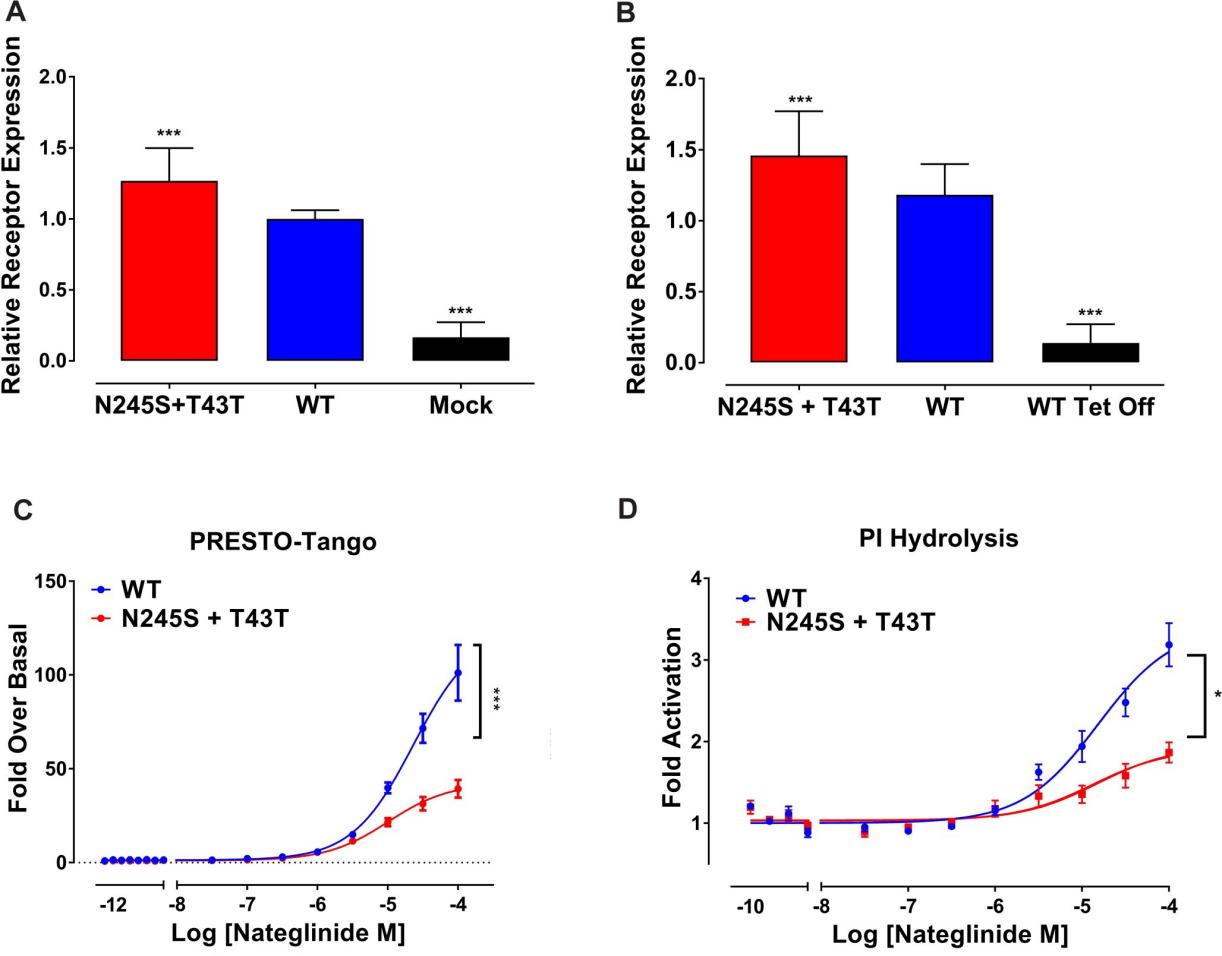
MRGPRX4 N245S variant has dampened signaling when compared to WT. (A) Receptor expression as calculated by whole cell ELISA in HTLA cells transfected with N245S+T43T or WT receptor with mock transfected shown as negative control (n = 2, 64 wells per experiment; y-axis is fold expression normalized to WT). (B) Receptor expression as calculated by whole cell ELISA in tetracycline inducible WT or N245S+T43T cells with non-tetracycline-induced cells shown as negative control (n = 2, 64 wells per experiment; y-axis depicts fold expression normalized to WT). (C) Average concentration response curves for Nateglinide in PRESTO-Tango arrestin assay with WT and N245S+T43T receptors, (n = 4, in quadruplicate; y-axis is in fold response over basal signaling). (D) Average concentration response curves for Nateglinide in PI hydrolysis assay with WT and N245S+T43T tet-inducible cell lines, (n = 3, in duplicate; y-axis is in fold response over basal signaling). * indicates P<0.05, *** indicates P<0.001 as calculated by F-test.

MRGPRX4 is an orphan G protein-coupled receptor (GPCR) expressed in mammalian sensory neurons [22, 23]. Although the endogenous ligand(s) for this receptor are not known, the potassium channel modulator Nateglinide has been identified to be a highly efficacious agonist and was used to demonstrate that this receptor couples predominantly to G_αq_ [26]. We used a cell-based approach to determine if the N245S variant affected the responsiveness of either MRGPRX4 β-arrestin or G_αq_ downstream signaling in response to Nateglinide. We first generated FLAG-tagged, codon-optimized wild-type (WT) and N245S variant constructs for the PRESTO-Tango β-arrestin recruitment assay and then generated stable, tetracycline-inducible cell lines for the FLAG-tagged WT MRGPRX4 and the N245S variant. Notably, all N245S variant constructs in this study also included the synonymous variant T43T. To measure membrane expression levels of WT MRGPRX4 and N245S+T43T, we used an established whole-cell ELISA assay [27] in HTLA cells. Using a 1-way ANOVA, we determined that N245S+T43T was expressed slightly but significantly more than the WT receptor in HTLA cells and in the tetracycline-inducible stable cells (Fig 2A and 2B).

We then examined the effect of the agonist Nateglinide on the recruitment of β-arrestin in the PRESTO-Tango recruitment assay, which provides a quantitative measure of receptor activation and downstream signaling [28, 29]. We found that Nateglinide had equal potency at both N245S+T43T and WT receptors, but the N245S+T43T variant displayed a dramatic reduction of fold activation (42 fold) in β-arrestin recruitment, significantly less than the WT receptor (123.9 fold) (P<0.001, Fig 2C, S6 Table). In an independent quantitative assay, we also examined the effect of the variant on G protein signaling using the G protein-dependent phosphatidylinositol (PI) hydrolysis assay. We observed that the maximal (E_max_) PI hydrolysis values following Nateglinide addition were significantly reduced in cells expressing the N245S+T43T variant compared with those expressing the WT receptor (PI Hydrolysis P<0.001, Fig 2D, S6 Table). Together, these data demonstrate that despite an apparent increase in N245S+T43T expression, the variant has significantly reduced arrestin and G protein signaling in comparison to WT.

### (-)-Menthol suppresses MRGPRX4 agonism

**Fig 3:**
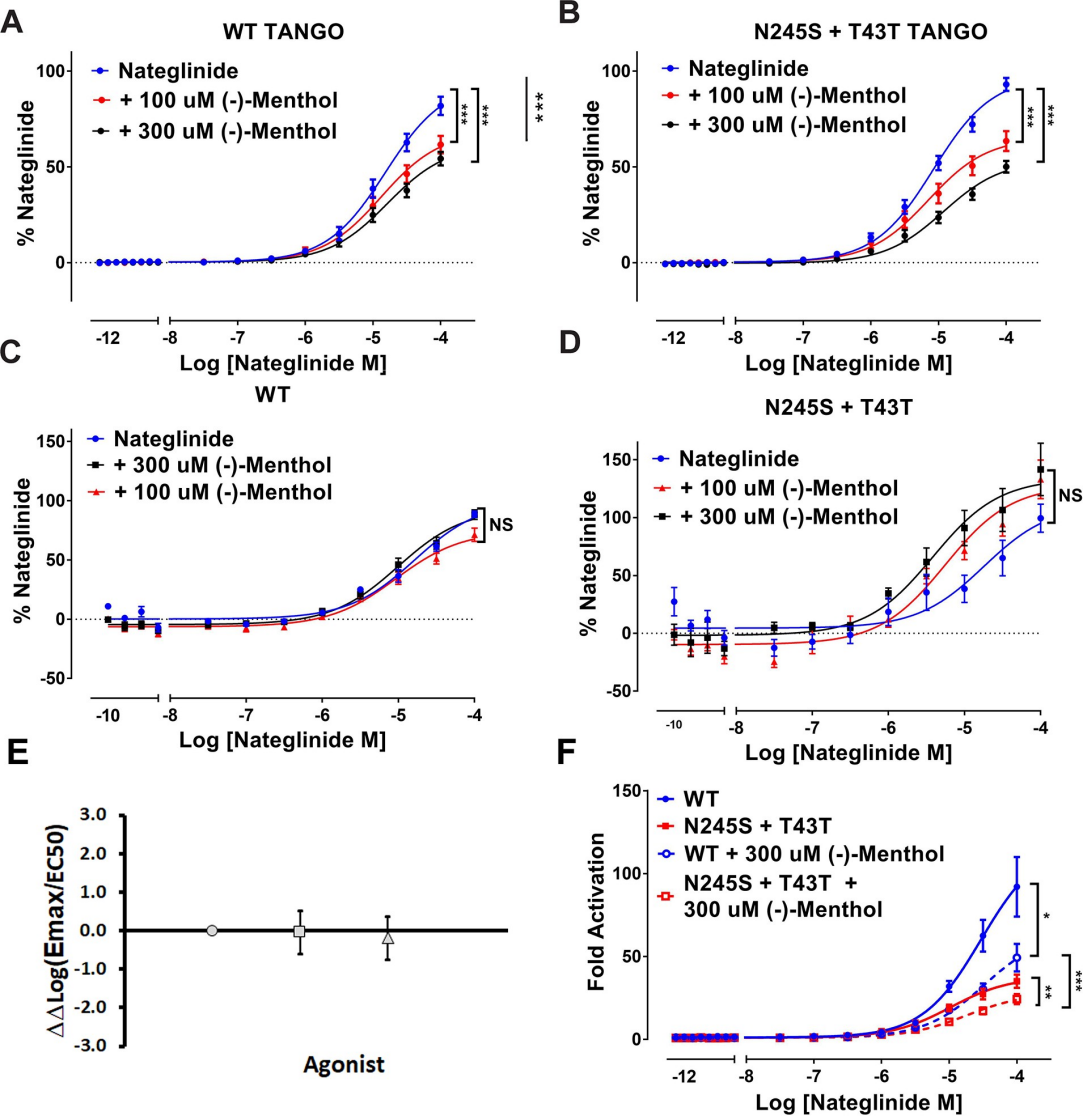
(-)-Menthol reduces MRGPRX4 WT and N245S+T43T arrestin signaling. (A),(B) Average concentration response curves for Nateglinide in MRGPRX4-WT-Tango (A) or MRGPRX4-N245S+T43T-Tango (B) following 100 μM or 300 μM (-)-menthol addition, (n = 3, in triplicate, y-axis is % Nateglinide). (C),(D) Average concentration response curves for Nateglinide-induced PI hydrolysis in MRGPRX4-WT (C) or MRGPRX4- N245S+T43T (D) tetracycline inducible cells following 100 μM or 300 μM (-)-menthol addition, (n = 3, in duplicate, y-axis is % Nateglinide). (E) Plot showing the effect of agonist treatments (x-axis) Nateglinide (gray circles), Nateglinide + 100 μM (-)-menthol (gray squares) and Nateglinide + 300 μM (-)-menthol (gray triangles) between WT and N245S+T43T variant shown as ΔΔLog(E_max_/EC_50_) values (y-axis). Y vales > 0 indicate increased effect for agonist at WT and values < 0 indicate increased effect for agonist at N+T variant. Bars depict 95% confidence intervals. (F) Average concentration response curves for fold change activation with Nateglinide in MRGPRX4-WT-Tango or MRGPRX4-N245S+T43T-Tango following 300 μM or 300 μM (-)-menthol addition, (n = 3, in quadruplicate). For all: NS = not significant, * p<0.05, ** p <0.01, and *** p<0.001 as calculated by F-test.

To determine whether (-)-menthol, the additive present in menthol cigarettes, alters the activity of MRGPRX4 WT or the N245S+T43T variant, we added the compound and repeated the functional assays. In the Tango assay, (-)-menthol alone showed no agonist activity at MRGPRX4 (up to 1 mM) (S3 Fig). We then tested whether (-)-menthol altered agonist-induced activity of the WT and N245S+T43T receptors. Increasing concentrations of (-)-menthol were added to each assay together with Nateglinide (Fig 3). We observed that 100 μM and 300 μM (-)-menthol significantly reduced the E_max_ of the agonist Nateglinide on the WT (P<0.001) and N245S+T43T (P<0.001) in the arrestin pathway (Fig 3A and 3B) but not in the G protein pathway as measured using the PI hydrolysis assay (Fig 3C and 3D). To determine whether (-)-menthol’s modulatory effect differed significantly between WT and N245S+T43T variant, we calculated ΔΔlog(E_max_/EC_50_) [30] for our reference agonist Nateglinide in the presence or absence of 100 and 300 μM (-)-menthol and found that (-)-menthol modulated WT and the variant equivalently (Fig 3E). A comparison of the fold change activation of β-arrestin recruitment revealed that 300 μM (-)-menthol significantly reduces Nateglinide-induced activation of the N245S variant when compared to WT (P<0.001, Fig 3F), similar to the differences in fold change for non-menthol conditions (Fig 2C). To test for non-specific effects of menthol on cells or cell membranes, we tested the effect of menthol on the unrelated D2 dopamine receptor in the PRESTO-Tango assay. This control showed that (-)-menthol had no modulatory effect on this receptor in this assay (S3 Fig, panel C). Similarly, (-)-menthol and Nateglinide had no effect on PI hydrolysis in cells where tetracycline was not added (i.e., with no MRGPRX4 receptor expression) (S3 Fig, panel D).

### Menthol affects agonism of the MRGPRX4 N245S receptor

**Fig 4:**
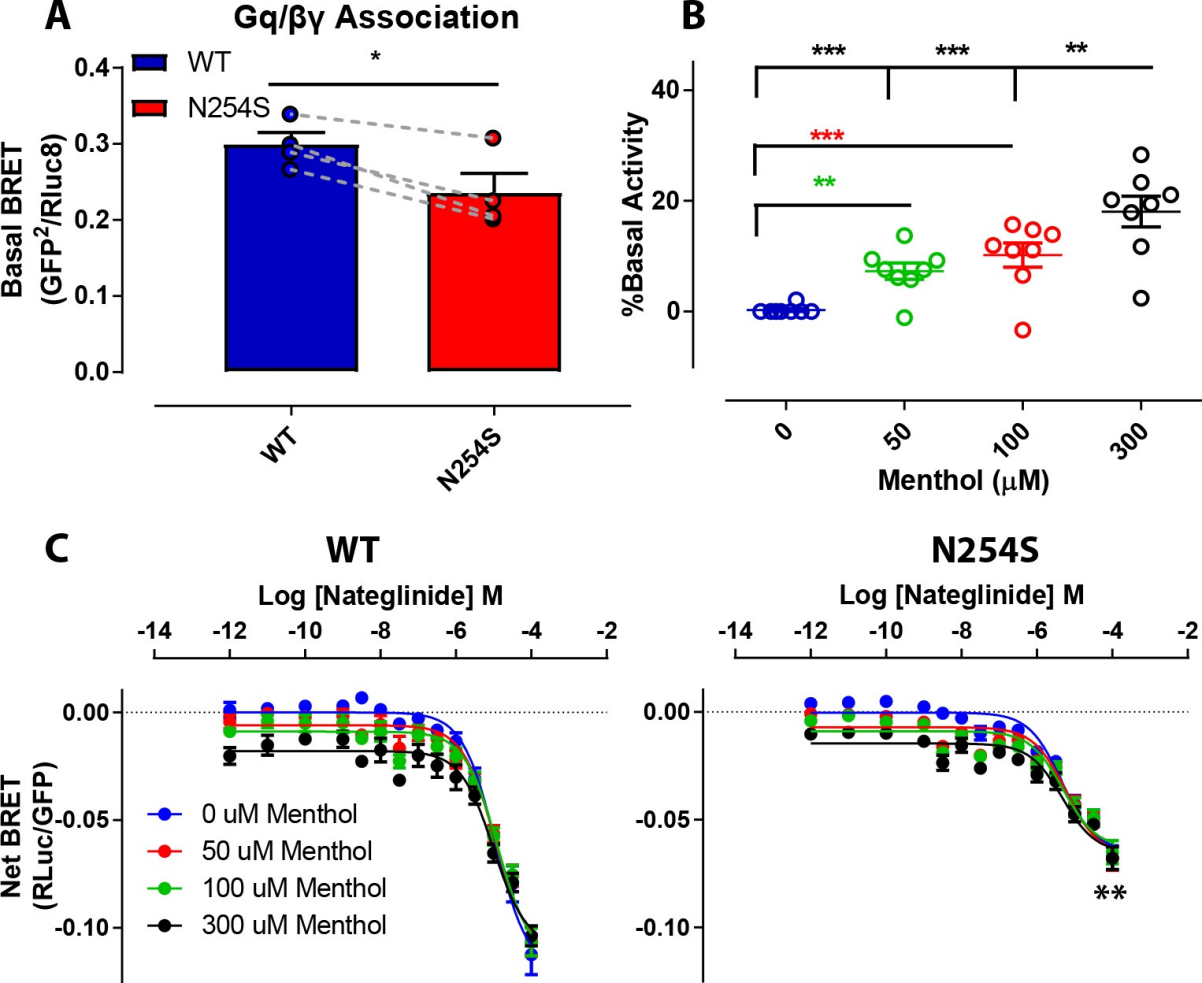
Bioluminesence resonance energy transfer (BRET) analysis of MRGPRX4 activity. (A). Differences in basal BRET activity of the WT and variant (N245S) MRGPRX4 receptors. (B). The effect of menthol on the basal activity of MRGPRX4. (C). The effects of varying concentrations of menthol on MRGPRX4 agonism by Nateglinide.

We also performed further studies using bioluminescence resonance energy transfer (BRET) as another measure of MRGPRX4 activity. Basal BRET in the G_q/βγ_ dissociation assay (an index of constitutive receptor activity) was reduced at MRGPRX4 N245S relative to WT (Fig 4A, 0.24 ± 0.025 vs 0.3 ± 0.015, t(3) = 4.986; *p* = 0.0155). Conversely, while basal activity of both WT and MRGPRX4 N245S was found to be increased by menthol (F(3,18) = 32.89, *p* < 0.0001), there was no effect of or interaction with genotype. Increasing amounts of menthol resulted in significantly elevated activity (Fig 4B, Linear effect, F(1,28) = 42.83; *p* < 0.0001) at all increments except between 50 and 100 μM (Fig 4B). Under agonist stimulation conditions, the probe Nateglinide showed an approximate 2-fold greater potency at MRGPRX4 N245S compared to WT (Table 5, EC50 values of 10.49 ± 0.61 μM vs 5.25 ± 0.25 μM). This effect was significant (F(1,18) = 7.44, *p* = 0.0343) accounting for 43.21% of the variance between the two populations. No effect of menthol on potency nor interaction with genotype were indicated. Similarly, efficacy of the probe Nateglinide (E_max_) was not affected by menthol at any concentration (Fig 4C), though E_max_ as calculated by net BRET was reduced at MRGPRX4 N245S vs WT (-0.067 ± 0.001 vs -0.113 ± 0.006, F(1,6) = 15.51, *p* = 0.0076).

**Table 5:** BRET measure of the effect of menthol on agonist potency.

		μM Menthol			
**EC50**	μM	0	50	100	300
WT	10.49 ± 0.69	14.23 ± 0.72	7.99 ± 1.01	11.71 ± 1.04	8.04 ± 1.91
N245S	5.25 ± 0.2548	2.01 ± 0.29	6.20 ± 0.74	7.60 ± 1.20	5.17 ± 0.99
**EMax**	Net BRET				
WT	-0.113 ± 0.006	-0.092 ± 0.002	-0.131 ± 0.009	-0.109 ± 0.001	-0.120 ± 0.004
N245S	-0.067 ± 0.001	-0.063 ± 0.001	-0.055 ± 0.001	-0.060 ± 0.001	-0.091 ± 0.001

### MRGPRX4 is expressed in the relevant sensory dorsal root ganglia

To provide further evidence for a role of MRGPRX4 in somatosensation, we used RT-PCR with RNA obtained from human thoracic dorsal root ganglia (DRG). The DRG serves to relay sensory information to the central nervous system, with the thoracic DRGs receiving sensory input from the lungs and airway. Using RT-PCR primers covering the length of *MRGPRX4*, following by sequencing of the RT-PCR products to ensure they originated from *MRGPRX4* rather than any of the other closely related *MRGPRX* genes, we found clear expression in this tissue (S4 Fig), consistent with a role for MRGPRX4 in somatosensation in tissues exposed to cigarette smoke.

## Discussion

We have identified a variant haplotype of *MRGPRX4* that is associated with increased prevalence of menthol cigarette smoking. This variant is found solely in individuals of African ancestry, and increases the odds of menthol use 5-to-8 fold among cigarette smokers. Cell-based assays of MRGPRX4 receptor function identified menthol as a novel negative modulator for this receptor, acting to reduce the responsiveness of this G protein-coupled receptor to its only known agonist at the WT and African-specific coding variant further.

While our understanding of *MRGPRX4* gene function is limited, the members of this gene family are expressed in primary sensory neurons and are believed to be involved in somatosensation and nociception, including pruritus [22–24]. Although the natural ligand(s) for MRGPRX4 have not yet been identified, it is of interest that the MRGPRX4 agonist Nateglinide, a drug used to treat Type 2 diabetes, has been reported to have pruritus as a side effect [31]. Together, this suggests that menthol may act outside of the taste sensory system and may exert an anesthetic effect, which is further enhanced by the African-specific form of this receptor, which has dampened signaling capacity.

The *MRGPRX4* variant associated with menthol cigarette smoking is relatively uncommon, with a MAF 8% in African Americans. Therefore, this variant alone cannot account for all of the difference in menthol cigarette smoking prevalence between African Americans and other ethnic groups. Thus, it is likely that other factors contribute to these differences. Surprisingly, we did not observe any consistent association at loci previously reported to be associated with menthol cigarette smoking (such as *TRPA1* and *TAS2R38*), or the gene encoding the TRPM8 channel, which has been shown to be the target for menthol action in the somatosensory system. This may be due to the small size of our discovery cohort, which was powered to discover only large effect sizes (OR >2–3). The *TRPA1* variants previously linked to menthol smoking had more modest effect sizes (odds ratios 1.3–1.4); thus, our study may have had insufficient power to detect their effects. We also genotyped the *TRPA1* SNPs in our Schroeder cohort (N = 741) and could not replicate these associations, suggesting that factors other than power may be responsible for the difference in the results. The previously described association of *TRPA1* variants with menthol smoking was restricted to heavy smokers, and was not observed in lighter smokers. Although our discovery cohort likely included a substantial proportion of light smokers, our replication cohort included mostly heavy smokers, which suggests that the lack of association in *TRPA1* is unlikely to be explained entirely by the difference in phenotype.

Another possibility is that menthol smoking preferences are regulated by *TRPA1* or *TRPM8* non-coding variants that were not captured by the Exome chip or whole-exome sequencing. However, we have previously sequenced the exons and adjacent intronic regions of *TRPM8* and *TRPA1* in the Schroeder and Dallas cohorts and could not find variants with a consistent association with menthol smoking. Nevertheless, these genes remain plausible candidates and further studies, including larger samples of precisely phenotyped individuals, are warranted.

There are currently no crystal structures of the MRGPRX4 receptor available to conclusively determine the location and role of the N245S variant uncovered in this study. Based on a sequence alignment of MRGPRX4 with a published computational model of the related receptor MRGPRX2 [32], N245 (D in MRGPRX2) appears to be located in the third extracellular loop (EL3) of the receptor. Here, N245S reduces Nateglinide-induced agonism in both arrestin and G protein signaling pathways despite an apparent increase in membrane expression. EL3 has been demonstrated in the serotonin receptor 5HT2B to be involved in sterics of ligand binding and the kinetics of ligand and receptor interactions [28]. Thus, it is possible that the N245S variant changes the steric or kinetic properties of Nateglinide binding that influence arrestin and G protein signaling, though further studies will be needed to dissect the mechanism of this effect.

The strengths of our study are the use of a population-based sample including both African-American and European-American smokers, and a replication in a large independent cohort of African Americans. Unlike previous studies that looked at candidate polymorphisms, we performed a hypothesis-free exome-wide screen that provided broad and dense coverage of variation in the coding regions of the genome.

One limitation of our study, as mentioned above, is the relatively small size of our discovery cohort, which provided adequate power to discover only variants with large effect sizes (OR>2–3), and may have missed other genetic variants with lower allele frequencies or smaller effect sizes. Nevertheless, our approach represents an unbiased investigation into the genetic determinants of menthol cigarette smoking in a multiethnic cohort. Another potential limitation is that our data on menthol cigarette use was based on self-report, thus some individuals may have been misclassified with regard to their phenotype. However, our estimates of prevalence of menthol cigarette smoking among ethnic groups were consistent with national estimates, suggesting that misclassification error, if present, is likely small. Likewise, if such misclassification in the Schroeder population led to an overestimate of the association between *MRGPRX4* and menthol smoking due to hidden population substructure, this is likely to be small because we found minimal evidence for heterogeneity within this group by large-scale SNP genotyping. Finally, not all participants responded to our questionnaire, thus results may not generalize to other populations. However, responders were similar to non-responders in terms of age and ethnicity (see Materials and Methods).

Menthol is known to exert its effects through transient receptor potential (TRP) channels TRPM8 [33, 34], and to a lesser extent TRPA1 [14]. TRPM8 is also known to mediate menthol-induced analgesia [35–37], and studies have shown that even low levels of menthol in tobacco, below those required to produce mint-like taste or aroma in tobacco, can activate TRPM8 [38]. Although our study was underpowered to detect variants with small effects, we found no evidence of association between variants at the *TRPM8* and *TRPA1* loci and menthol use, suggesting that variation in these menthol receptors is not a major contributor to the differential use of menthol cigarettes among African Americans.

Menthol cigarettes have been identified as a major threat to public health that have a disproportionate effect on ethnic minorities [39]. Our data suggest that ancestry-specific variants in genes involved in nociception contribute to both inter-individual and inter-ethnic differences in menthol cigarette smoking. The existence of population-specific genetic variants presents a new risk factor for menthol cigarette use, and suggests that the existence of this risk factor can inform health policies and tobacco regulatory actions designed to reduce health disparities in the United States.

## Materials and methods

### Ethics statement

Participants gave written informed consent under protocols approved by the IRB of the University of Texas Southwestern Medical Center (protocol #STU 112013–048), and the Western IRB (protocol #20131296). The overall study was carried out under protocol #01-DC-0230 and was approved by National Institutes of Health Combined Neurosciences IRB.

### Study populations

#### Discovery cohort

For the primary analysis, we enrolled participants from two population-based cohorts: the Dallas Heart Study (DHS) and the Dallas Biobank. DHS is a multiethnic population-based probability sample of Dallas County, Texas, with intentional oversampling of African Americans [40]. All participants completed a detailed survey and underwent a physical examination, including collection of blood samples for laboratory and genetic analyses. Race and ethnicity were self-assigned according to categories used in the US Census. Smoking status was obtained from the questionnaire. A total of 2267 individuals (including 1217 African Americans self-identified as non-Hispanic black, 720 European Americans self-identified as non-Hispanic white, and 289 Hispanics) identified themselves as current of former smokers and were eligible for the present study. Due to the small number of Hispanic smokers, we limited our investigation to African-American and European-American participants. The Dallas Biobank is a repository of DNA and plasma samples from individuals ascertained at various locations in north-central Texas. Smoking status was obtained via questionnaire. African-American individuals who identified themselves as smokers (N = 2601) were eligible for the present study. All participants were ≥18 years of age.

#### Menthol data collection

All eligible participants were invited to complete a survey regarding mentholated tobacco use, via telephone and/or email. The survey was approved by the IRB of the University of Texas Southwestern Medical Center under protocol number STU 112013–048. A total of 677 individuals (including 438 DHS and 239 Biobank participants) completed the questionnaire. Responders were more often female compared to non-responders (58.7% versus 49.0% in DHS, and 67.4% versus 52.9% in Biobank, P<0.001 for both), but were not significantly different from non-responders in age (mean age 50.2 ± 11.3 (SD) versus 49.8 ± 9.6 years in DHS, P = 0.45; and 43.6 ± 13.6 versus 43.6 ± 13.0 years in Biobank, P = 0.93) or ethnic composition (63% versus 61% were African American in DHS, P = 0.39; 100% were African American in Biobank). Of the subjects who had completed the questionnaire, 561 (428 from DHS and 133 from Biobank) had exome-wide genotype data available (see below) and were included in our discovery cohort.

#### Replication cohort

To confirm the findings in the Dallas cohorts, we recruited participants from the Schroeder population, comprising a total of 741 Washington DC resident smokers who were enrolled through the DC Tobacco Quitline (DCQL) [41]. All subjects were self-described African Americans, aged ≥18 years. General variables, such as gender, cigarettes per day (CPD), marital status and education level were obtained from all subjects. Data on mentholated cigarette use were obtained via a questionnaire and from the Wisconsin Inventory of Smoking Dependence Motives (WISDM) [42].

### Genotyping

In the Dallas cohorts, genomic DNA was extracted from circulating leukocytes. A total of 4,591 DHS participants and 4,975 African-American participants from the Biobank were previously genotyped using Illumina Infinium HumanExome BeadChip v12.1, which captured >200K markers, including protein-altering variants (>90%), disease-associated variants from previously published genome-wide association studies, ancestry-informative markers, and other variants. Genotypes were called using Illumina GenomeStudio software. Samples were excluded if they met the following criteria: genotype call rate <99%, duplicate sample, discordant duplicate pair, or genotyped gender did match the stated gender. Variants were excluded based on a call rate of <99% or a deviation from Hardy-Weinberg equilibrium in African Americans with P <0.0001. Of the individuals who were successfully genotyped, 561 had data on menthol cigarette smoking and were included in the present study. After quality filtering, 116,212 autosomal variants were polymorphic in our study sample. Due to the relatively small sample size, we removed variants with MAF<1% (<10 carriers). After exclusions, 52,298 autosomal variants were available for analysis.

In the Schroeder population, DNA was collected using Oragene saliva collection kits and extracted according to the manufacturer’s protocol (Genotek Inc., Kanata, Ontario, Canada). Variants identified in the Dallas cohort were assayed by Sanger sequencing, using a dedicated set of primers (S2 Table). DNA chromatograms were analyzed and checked individually in order to evaluate the presence of calling errors with the Lasergene suite (DNASTAR, Madison, Wisconsin). In addition, 24 menthol smokers and 24 non-menthol smokers were randomly chosen and genotyped using the Illumina HumanOmni1 Chip that assayed 1,140,419 SNPs genome-wide to estimate ancestry levels. Variants were excluded based on a call rate of <99% or a deviation from Hardy-Weinberg equilibrium with P <0.0001. High-quality variants were further pruned for linkage disequilibrium (r^2^<0.1). Ancestry was inferred using ADMIXTURE software v.1.3.0 [43].

### Whole-exome sequencing

Exons of *MRGPRX4* were sequenced in a subset of Dallas participants (N = 389) using whole-exome sequencing, as part of a related investigation into the role of genetic variation in smoking behaviors. Sample preparation and whole-exome sequencing were performed at the McDermott Center Next-Generation Sequencing core. Three micrograms of genomic DNA was sonicated using a Covaris S2 ultrasonicator (Covaris, Woburn, MA), purified, and assessed using an Agilent 2100 Bioanalyzer (Agilent Technologies, Santa Clara, CA). DNA was end-repaired, and 3′ ends were adenylated and barcoded with truncated adapters. PCR amplified libraries were purified with AmpureXP beads (New England Biolabs, Ipswich, MA) and assayed using an Agilent 2100 Bioanalyzer. A 750 ng aliquot of the fragment library was concentrated by vacuum to 3.5 μL and hybridized and captured with a SureSelect Human All Exon V4 kit (Agilent Technologies, Santa Clara, CA). Following hybridization, the captured library was amplified and index tags were added to the adapters. DNA was again purified with AmpureXP beads, and fragment sizes were assayed using the Agilent 2100 Bioanalyzer. Paired-end sequencing (150 basepairs) was performed using an Illumina Hiseq 2500. We achieved sufficient coverage depth to provide mean coverage of >115x for targeted bases, with over 94% of target bases covered at least 20x in 95% of the samples (>91% of target bases covered ≥20x in >99% of the samples).

Reads were aligned to the human reference genome build GRCh37 using the Burrows-Wheeler Alignment Tool [44], and variants were called using the Genome Analysis Toolkit (GATK) HaplotypeCaller [45]. Only high-quality variants (GQ >80% and allele depth >20x) were retained for analysis. Variants were annotated using SnpEff [46].

### Exome-wide association analysis

In the Dallas cohorts, ancestry was inferred using principal component analysis implemented in EIGENSTRAT [47]. Exome-wide association analysis was performed using PLINK v1.90p [48]. Significance was determined based on a likelihood-ratio test [49], using an R [50] plug-in function for logistic regression. An additive genetic model was assumed, and the analysis was adjusted for age, gender, and 6 leading principal components of ancestry. All variants reaching a significance threshold P<1x10^-4^ in the discovery cohort were tested for replication in the validation cohort. The analysis was performed in PLINK, with adjustment for age and gender. The association results were combined using random-effects inverse-variance weighted meta-analysis.

### Dorsal root ganglia expression

Fresh frozen human dorsal root ganglia was obtained from the National Disease Research Interchange (https://ndriresource.org) under protocol DDRD6 001 001. Following tissue preparation on a Covaris CPO2/S2, RNA was purified using RNeasy Quick start (Qiagen) according to the manufacturer’s instructions and including a DNase digestion. The resulting total RNA was used as template for cDNA synthesis using Superscript IV First-Strand Synthesis System (Invitrogen) with the Oligo d(T)_20_ primers. Subsequent PCR primers (S8 Table) were used to generate PCR products that were analyzed on a 1% agarose gel (S4 Fig), followed by dideoxy- Sanger sequencing to confirm the RT-PCR products originated from *MRGPRX4*, rather than the other, closely related *MRGPRX* gene family members.

### Functional studies

#### Cells and reagents

HTLA cells were a gift from Dr. Richard Axel (Columbia University) and were maintained at low passage batches in DMEM (Corning) containing 10% FBS, 2 μg/mL puromycin and 100 μg/mL hygromycin B in a humidified atmosphere at 37°C with 5% CO_2_. Inducible MRGPRX4 and MRGPRX4 mutant stable cell lines were generated according to manufacturer’s instructions from the FLP-IN/T-REX HEK-293 cells (Invitrogen, Cat# R78007) and were certified as HEK and mycoplasma-free by Invitrogen.

#### Constructs

MRGPRX4-Tango codon-optimized plasmids were made as previously described [26]. The MRGPRX4 receptor was then subcloned into the FLP-IN construct without the Tango C-terminus. Mutant MRGPRX4-N245S, MRGPRX4-T43T, and MRGPRX4-N245S+T43T constructs were generated by site-directed mutagenesis PCR using PrimeStar (Takara). Mutations were confirmed by Sanger sequencing using primers V2tail forward, CMVforward BGHR, and TEV tail reverse primers.

#### Whole cell ELISA

To compare surface expression of the FLAG-tagged MRGPRX4 receptor and the MRGPRX4 mutant N245S, we performed immunohistochemistry on whole, unpermeabilized cells plated in 384-well plates as previously described [27]. Briefly, transfected cells or stably expressing MRGPRX4 cells were seeded at a density of 10,000 cells/well in poly-lysine-coated 384 well plates. After 16–18 hours, cells were fixed with 20 ul/well of 4% paraformaldehyde solution for 10 minutes at room temperature. Cells were then washed twice with 40 ul/well of phosphate-buffered saline (PBS), pH 7.4. Cells were then blocked with 5% normal goat serum in PBS for 30 minutes at room temperature. After removing blocking solution, 20 ul/well of anti-FLAG–horseradish peroxidase–conjugated antibody (Sigma-Aldrich, diluted 1/10,000) was added and incubated at room temperature for 1 hour. Following antibody incubation, cells were washed 2x with 80 ul/well of PBS. Then, 20 ul/well of SuperSignal Enzyme-Linked Immunosorbent Assay Pico Substrate (Sigma-Aldrich) was added and the resulting luminescence was measured using a MicroBeta Trilux luminescence counter. Replicates were averaged by taking fold change compared to WT receptor expression. Each experiment was performed with 64 wells per receptor.

#### Presto-tango β-arrestin assay

In brief, HTLA cells were seeded at 50% confluency and transfected with MRGPRX4-Tango constructs using the calcium phosphate method [51]. The following day, transfected cells were transferred to glass-bottomed, poly-L-lysine-coated white 384-well plates at a density of 20,000 cells/well in DMEM (Corning) supplemented with 1% dialyzed FBS and 100 IU/mL penicillin and 100 μg/ml streptomycin. The next day, cells were treated with concentration response curves in triplicate with MRGPRX4 agonist Nateglinide in the presence or absence of various concentrations of (-)-Menthol and incubated for 18–24 hours. All chemical compounds were diluted in drug buffer (1X HBSS with 20 mM HEPES and 0.3% Bovine Serum Albumin, pH 7.4). After drug incubation, medium was removed, 20 μL/well of Bright Glo (Promega) (diluted 20-fold) was added, and luminescence was measured on a TriLux luminescence counter. Replicates were averaged using normalized data (fold change over basal).

#### Inositol phosphate hydrolysis

MRGPRX4 tetracycline-inducible cells were maintained in DMEM containing 10% FBS, 100 μg/mL hygromycin B, and 15 μg/mL blasticidin. For the assay, cells were seeded into poly-L-lysine coated, glass-bottom 96 well plates at a density of 50,000 cells/well in inositol-free DMEM (Caisson labs) containing 1 μCi/well of ^3^H-myo-inositol (Perkin Elmer), 1 μg/mL tetracycline, and 100 IU/mL penicillin and 100 μg/ml streptomycin and incubated 16–18 hours in a humidified environment at 37°C with 5% CO_2._ Following tetracycline induction and labeling, medium was removed and 200 μl/well of inositol-free DMEM (Caisson labs) was added per well. Then, 25 μl of 10X concentrated (-)-menthol diluted in drug buffer (1X HBSS with 20 mM HEPES and 0.3% bovine serum albumin, pH 7.4) was added followed by 25 μl of 10X concentrated Nateglinide in concentration response curve. Cells were incubated with drug for 1 hour in a humidified environment at 37°C with 5% CO_2._ At exactly 15 minutes before lysing, 10 μl/well of 26X concentrated LiCl (15 mM final concentration) was added. After drug and LiCl incubation, 40 μl/well of 50 mM formic acid was added and cells were incubated at 4°C overnight. The next day, 10 μl of lysate was transferred from each well into 96-well flexi-plates (Perkin Elmer) and combined with 75 μl/well of 0.2 mg/well YSI RNA Binding Beads (Perkin Elmer). Lysate-bead mixture was incubated on a shaker at room temperature, protected from light, for 1 hour. Before reading on a TriLux beta counter, plates were centrifuged at 1000 x g for 2 minutes. Replicates were averaged using normalized data (fold change over basal).

#### BRET assay

Using Transit-2020, HEK293T cells were transfected with 1.5 μg Gαq-rLuc8, 1.5 μg γ1-GFP, 1.5 μg Gβ1 and 1.5 μg of either MRGPRX4 WT or MRGPRX4 N245S. The next day, cells were plated in 1% dFBS/DMEM at a cell density of 40–50,000 cells/well in white 96-well clear-bottom plates. On the third day, media was aspirated from the wells and cells were washed with 60 μL assay buffer (1x Hanks Buffered Saline Solution, 20 mM HEPES, pH 7.4). After the wash was aspirated, 60 μL of assay buffer was added to each well followed by 10 μL of 50 μM coelenterazine 400a. After 5 minutes, drugs were added and the plate was read on an LB940 Mithras using 405/515 emission filters at 1 second signal integration. Data were reported as the ratio of GFP-signal to luciferase signal.

### Statistical analysis

Baseline characteristics of participants were compared using t-tests or analysis of variance for continuous variables and chi-square tests for categorical variables. Analyses were performed using R v3.2.1 statistical analysis software [50]. Presto-tango assay data were analyzed in GraphPad Prism V6.07 using an F-test for each EC_50_ and Emax parameters. ΔΔlog(Emax/EC50) plots were calculated as described in Kenakin et al [30]. BRET assay data were analyzed in Graphpad Prism 7.0 using non-linear regression models. Net BRET was calculated by subtracting values from the no-drug condition. Data comparing effects of menthol were analyzed as two-way repeated measures ANOVA (n = 4 biological replicates, 2 technical replicates for each condition per plate). Post-hoc analyses were performed using a Tukey correction for multiple comparisons.

## Supporting information

S1 Fig Quantile-Quantile plot of -log_10_ p-values from exome-wide association analysis. (PDF)

S2 Fig Genetically inferred ancestry of Schroeder participants relative to populations represented in the 1000 genomes project. The figure shows that Schroeder subjects (SCHR) show a greater degree of European admixture (CEU, Utah Residents with Northern and Western Ancestry), relative to West African populations (YRI) and African American from Southwest US (ASW).
(PDF)

S3 Fig Controls indicating no significant effect of (-)-menthol on functional activity assays. (A),(B) Average concentration response curves for Nateglinide or (-)-menthol in agonist mode for MRGPRX4-WT-Tango or MRGPRX4- N245S+T43T-Tango (n = 2, in triplicate, y-axis is % Nateglinide). (C) Average concentration response curves for the dopamine D2-receptor agonist quinpirole in D2-Tango 100 μM or 300 μM (-)-menthol addition, (n = 3, in triplicate, y-axis is % Quinpirole). (D) Average concentration response curves for Nateglinide-induced PI hydrolysis in MRGPRX4-WT tetracycline inducible cells without tetracycline addition (i.e., no receptor expression) following 100 μM or 300 μM (-)-menthol addition, (n = 3, in triplicate, y-axis is relative luminescent counts (RLU).
(TIF)

S4 Fig RT-PCR of MRGPRX4 in human dorsal root ganglion tissue. (PDF)

S1 Table Association of *MRGPRX4* rs7102322 and menthol smoking stratified by gender. (DOCX)

S2 Table Primers used for Sanger sequencing in the Schroeder cohort. (DOCX)

S3 Table Primers used for genotyping *MRGPRX4* SNPs. (DOCX)

S4 Table Genotyping summary of ancestry-informative SNPs at the *MRGPRX4* locus. (DOCX)

S5 Table Associations between ancestry-informative *MRGPRX4* SNPs and menthol smoking. (DOCX)

S6 Table Statistics for WT versus variant values in PRESTO-Tango assays. (DOCX)

S7 Table Functional assays of WT and N245S+T43T MRGPRX4 variants. Part 1. Comparison of WT versus variant values. Part 2. Comparison of WT versus variant menthol response.
(DOCX)

S8 Table Primer sequence for *MRGPRX4* RT-PCR and sequencing of PCR products. (DOCX)
