# Supplementary figures and images for: An African-specific haplotype in MRGPRX4 is associated with menthol cigarette smoking
Source: PLoS Genet. 2019 Feb 15;15(2):e1007916. doi: 10.1371/journal.pgen.1007916 (PMC6377114; doi:10.1371/journal.pgen.1007916)

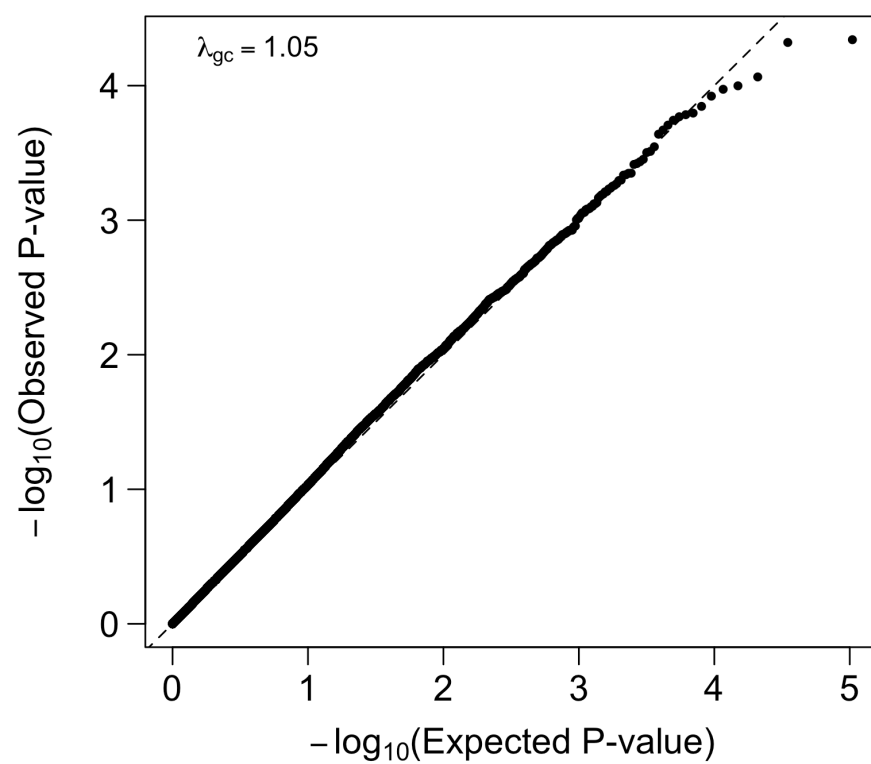

**S1 Figure.** Quantile-Quantile plot of  $-\log_{10}$  p-values from exome-wide association analysis.

Supplement: S1 Fig — (PDF) [file pgen.1007916.s001.pdf]

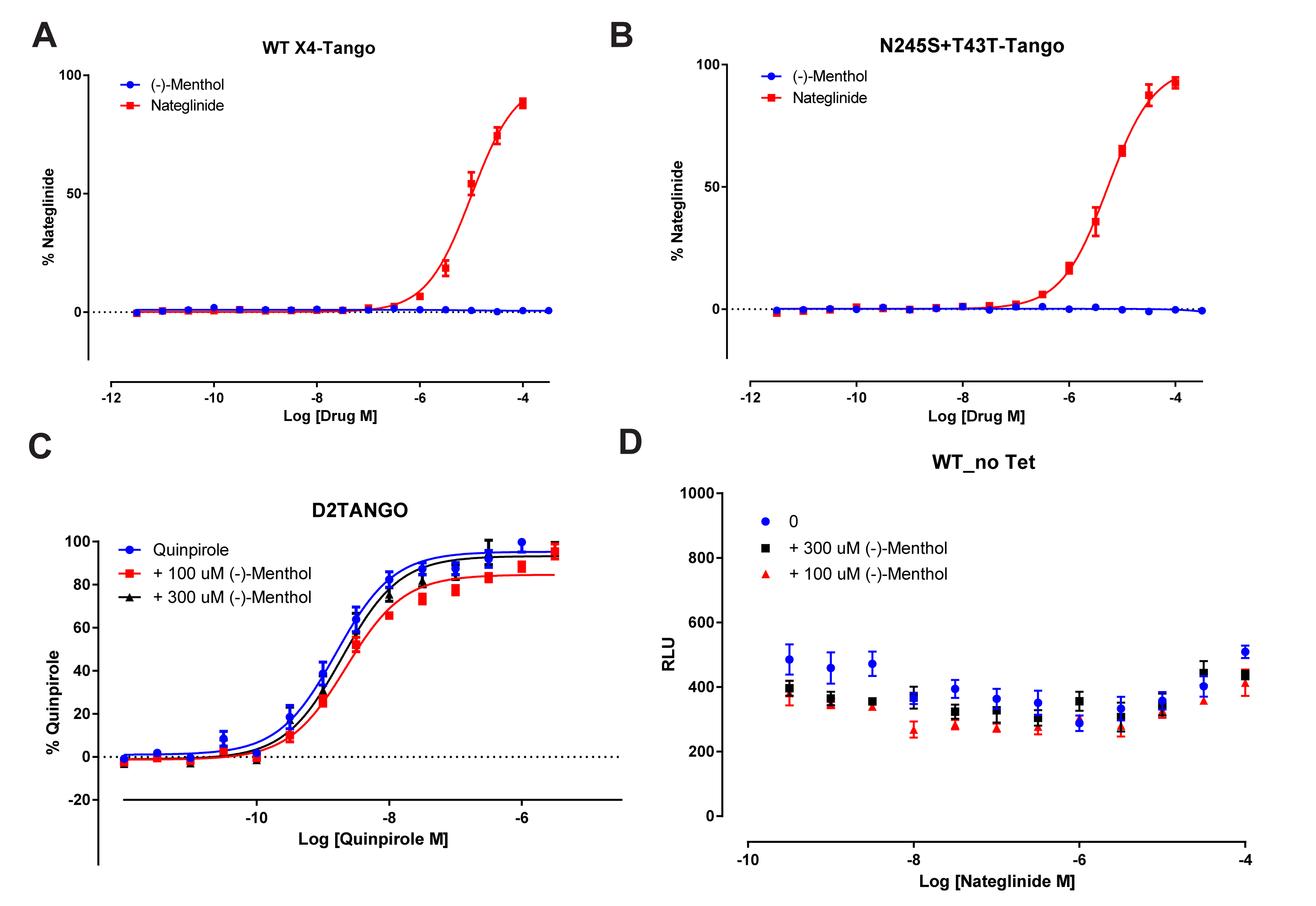

Supplement: S3 Fig — (A),(B) Average concentration response curves for Nateglinide or (-)-menthol in agonist mode for MRGPRX4-WT-Tango or MRGPRX4- N245S+T43T-Tango (n = 2, in triplicate, y-axis is % Nateglinide). (C) Average concentration response curves for the dopamine D2-receptor agonist quinpirole in D2-Tango 100 μM or 300 μM (-)-menthol addition, (n = 3, in triplicate, y-axis is % Quinpirole). (D) Average concentration response curves for Nateglinide-induced PI hydrolysis in MRGPRX4-WT tetracycline inducible cells without tetracycline addition (i.e., no receptor expression) following 100 μM or 300 μM (-)-menthol addition, (n = 3, in triplicate, y-axis is relative luminescent counts (RLU). (TIF) [file pgen.1007916.s003.tif]

**S5 Figure. RT-PCR of MRGPRX4 in human dorsal root ganglion tissue.**

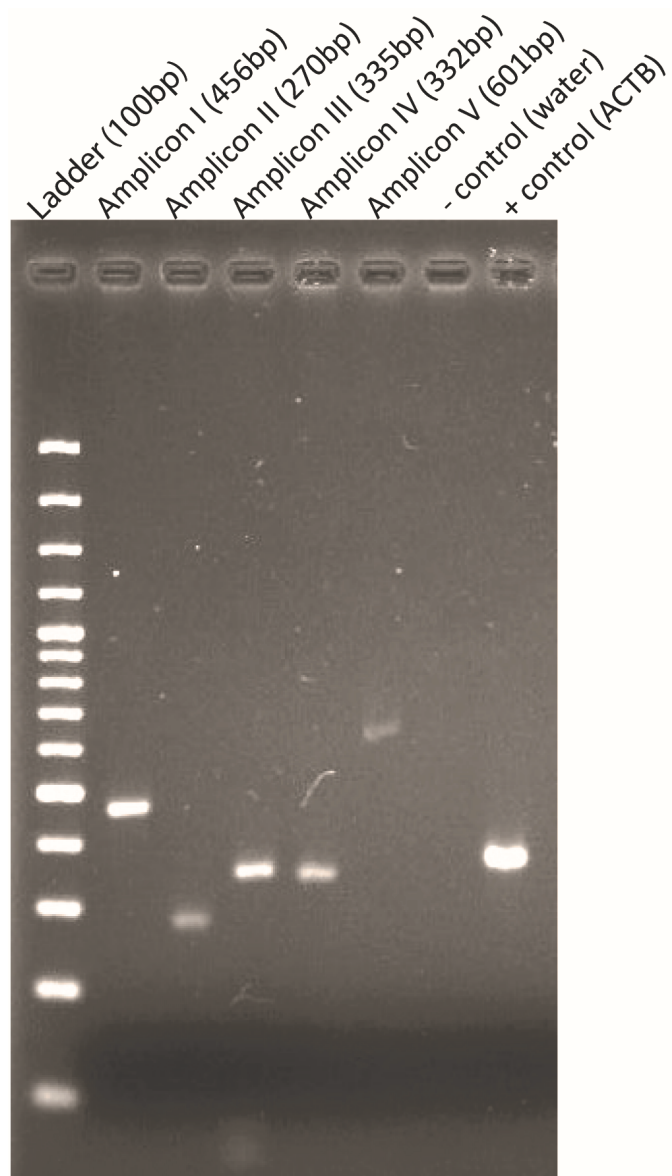

Supplement: S4 Fig — (PDF) [file pgen.1007916.s004.pdf]
