# Supplementary material for: An African-specific haplotype in MRGPRX4 is associated with menthol cigarette smoking
Source: PLoS Genet. 2019 Feb 15;15(2):e1007916. doi: 10.1371/journal.pgen.1007916 (PMC6377114; doi:10.1371/journal.pgen.1007916)
Supplement: S2 Fig — The figure shows that Schroeder subjects (SCHR) show a greater degree of European admixture (CEU, Utah Residents with Northern and Western Ancestry), relative to West African populations (YRI) and African American from Southwest US (ASW). (PDF) [file pgen.1007916.s002.pdf]

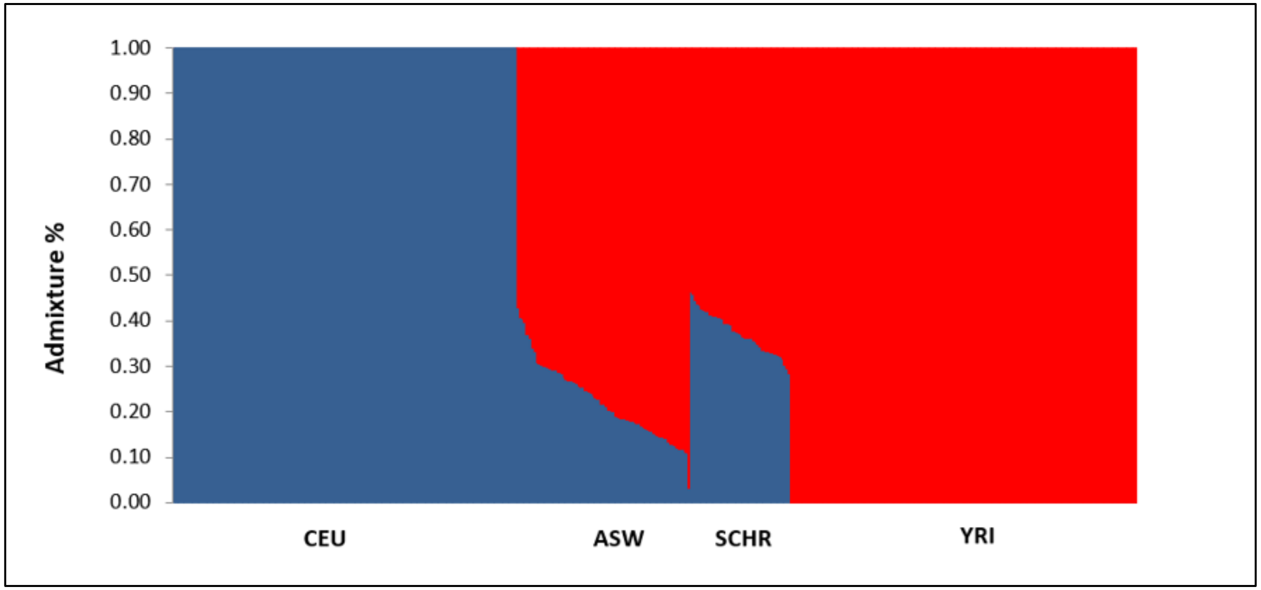

S2 Figure. Genetically inferred ancestry of Schroeder participants relative to populations represented in the 1000 genomes project. The figure shows that Schroeder subjects (SCHR) show a greater degree of European admixture (CEU, Utah Residents with Northern and Western Ancestry), relative to West African populations (YRI) and African American from Southwest US (ASW).
