# Supplementary material for: An African-specific haplotype in MRGPRX4 is associated with menthol cigarette smoking
Source: PLoS Genet. 2019 Feb 15;15(2):e1007916. doi: 10.1371/journal.pgen.1007916 (PMC6377114; doi:10.1371/journal.pgen.1007916)
Supplement: S1 Table — (DOCX) [file pgen.1007916.s005.docx]

| **Table S1. Association of MRGPRX4 rs7102322 and menthol smoking stratified by gender** | | | | | | | |
| --- | --- | --- | --- | --- | --- | --- | --- |
| **Cohort** | **Smoking** | **rs7102322 genotype** | | |  | **Allelic** | **P-value** |
|  | **Status** | **A/A** | **A/G** | **G/G** | **MAF** | **OR (95% CI)** |  |
| Dallas AA, Men | Non-Menthol, N | 29 | 2 | 0 | 3.2% |  |  |
|  | Menthol, N | 96 | 24 | 1 | 10.7% | 3.88 (0.83-18.09) | 0.046 |
|  | Menthol, % | 76.8% | 92.3% | 100.0% |  |  |  |
|  |  |  |  |  |  |  |  |
| Dallas AA, | Non-Menthol, N | 46 | 0 | 0 | 0.0% |  |  |
| Women | Menthol, N | 159 | 34 | 3 | 10.2% | Inf (0 - Inf)* | 6.4E-05 |
|  | Menthol, % | 77.6% | 100.0% | 100.0% |  |  |  |
|  |  |  |  |  |  |  |  |
| Schroeder, Men | Non-Menthol, N | 234 | 6 | 0 | 1.3% |  |  |
|  | Menthol, N | 219 | 37 | 0 | 7.2% | 6.56 (2.71-15.85) | 2.9E-05 |
|  | Menthol, % | 48.3% | 86.0% | - |  |  |  |
|  |  |  |  |  |  |  |  |
| Schroeder, Women | Non-Menthol, N | 75 | 2 | 0 | 1.3% |  |  |
|  | Menthol, N | 146 | 22 | 0 | 6.5% | 5.68 (1.30-24.81) | 0.021 |
|  | Menthol, % | 66.1% | 91.7% | - |  |  |  |
|  |  |  |  |  |  |  |  |
| MAF - minor allele frequency  P-values were calculated using logistic regression models adjusted for age. In the Dallas cohorts, the p-values are based on a likelihood ratio test, due to low numbers of carriers among non-menthol smokers. In the Schroeder cohort, the p-values are calculated using the Wald test. *Odds ratio could not be calculated in the Dallas females, because none of the variant carriers were non-menthol smokers. | | | | | | | |
