# Supplementary material for: An African-specific haplotype in MRGPRX4 is associated with menthol cigarette smoking
Source: PLoS Genet. 2019 Feb 15;15(2):e1007916. doi: 10.1371/journal.pgen.1007916 (PMC6377114; doi:10.1371/journal.pgen.1007916)
Supplement: S2 Table — (DOCX) [file pgen.1007916.s006.docx]

**Table S2: Primers Used for Sanger Sequencing in the Schroeder Cohort**

| **PrimerID** | **Gene** | **Direction** | **Sequence** | **Reaction** |
| --- | --- | --- | --- | --- |
| UpstreamF | MRGPRX4 | Forward | AGTCCTGTCTGCAATGACTGA | PCR+SEQ |
| UpstreamR | MRGPRX4 | Reverse | ACCGTGAAGCTCAGGGTCT | PCR+SEQ |
| UpstreamSeqF | MRGPRX4 | Forward | TTCCCATGTCAGCACAGAAC | SEQ |
| UpstreamSeqR | MRGPRX4 | Reverse | ATCTCTGATTCTCCCCACCA | SEQ |
| ORF1stHF | MRGPRX4 | Forward | GTGGTGGGGAGAATCAGAGA | PCR+SEQ |
| ORF1stHR | MRGPRX4 | Reverse | CACCAACTAGAATCAGCACCA | PCR+SEQ |
| ORF1stHSeqF | MRGPRX4 | Forward | TTCCTCTTCCTCAGCTTCCA | SEQ |
| ORF1stHSeqR | MRGPRX4 | Reverse | AGATGGGCCACAGAACAGAC | SEQ |
| ORF2ndHF | MRGPRX4 | Forward | GTCTGTTCTGTGGCCCATCT | PCR+SEQ |
| ORF2ndHR | MRGPRX4 | Reverse | AATGCTTTCCATGGGTGAAA | PCR+SEQ |
| ORF2FndHSeqF | MRGPRX4 | Forward | CAGTGCTGGTCTTCCTCCTC | SEQ |
| ORF2ndHSeqR | MRGPRX4 | Reverse | GAACCAGCTTCAGGTTCTGC | SEQ |
| ATR_1F | ATR | Forward | TGAATAAAAGTAGATGTTTCTTGTCCA | PCR+SEQ |
| ATR_1R | ATR | Reverse | AAACATTCAACCATAACAACGTATT | PCR+SEQ |
| PSDM3_1F | PSDM3 | Forward | GAATAGCATTATTGCTGATACGG | PCR+SEQ |
| PSDM3_1R | PSDM3 | Reverse | TCAGCACTTGCTGCTTCACT | PCR+SEQ |
