# Supplementary material for: An African-specific haplotype in MRGPRX4 is associated with menthol cigarette smoking
Source: PLoS Genet. 2019 Feb 15;15(2):e1007916. doi: 10.1371/journal.pgen.1007916 (PMC6377114; doi:10.1371/journal.pgen.1007916)
Supplement: S3 Table — (DOCX) [file pgen.1007916.s007.docx]

| **Table S3: Statistics for WT versus variant values** | | | | | | | | |
| --- | --- | --- | --- | --- | --- | --- | --- | --- |
| **Construct** | **Agonist** | **Modulator** | **EC50 (µM)** | **P** | **Emax (Fold)** | **P** | **Assay** | **Figure** |
| WT | Nateglinide | - | 23.02 | 0.096 | 123.90 | <0.001 | PRESTO-Tango | 1C |
| N245S+ T43T |  |  | 10.06 |  | 42.49 |  |  | 1D |
| WT | Nateglinide | - | 15.30 | 0.87 | 3.42 | 0.01 | PI Hydrolysis | 1D |
| N245S+ T43T |  |  | 13.33 |  | 1.92 |  |  | 1D |
|  |  |  |  |  |  |  |  |  |
| P values were calculated via F-test comparing statistical difference between either EC50 or Emax values between WT and variant. | | | | | | | | |
