# Supplementary material for: An African-specific haplotype in MRGPRX4 is associated with menthol cigarette smoking
Source: PLoS Genet. 2019 Feb 15;15(2):e1007916. doi: 10.1371/journal.pgen.1007916 (PMC6377114; doi:10.1371/journal.pgen.1007916)
Supplement: S4 Table — (DOCX) [file pgen.1007916.s008.docx]

T**able S4: Functional Assays of WT and N245S+T43T MRGPRX4 Variants**

| **Table S4, Part 1, Comparison of WT versus variant values** | | | |  |  |  |  |  |  |  |
| --- | --- | --- | --- | --- | --- | --- | --- | --- | --- | --- |
| **Construct** | **Agonist** | **Modulator** | **pEC50** | **SEM** | **P** | **Emax (Fold)** | **SEM** | **P** | **Assay** | **Figure** |
| WT | Nateglinide | - | -4.638 | 0.0846 | 0.10 | 123.90 | 8.52 | <0.001 | PRESTO-Tango | 2C |
| N245S+ T43T |  |  | -4.997 | 0.07299 |  | 42.49 | 1.93 |  |  | 2C |
| WT | Nateglinide | - | -4.814 | 0.1166 | 0.87 | 3.42 | 0.1944 | 0.01 | PI Hydrolysis | 2D |
| N245S+ T43T |  |  | -4.876 | 0.2372 |  | 1.92 | 0.1387 |  |  | 2D |
|  |  |  |  |  |  |  |  |  |  |  |
| **Table S4, Part 2, Compaiison of WT versus variant menthol response** | | | |  |  |  |  |  |  |  |
| **Construct** | **Agonist** | **Modulator** | **pEC50** | **SEM** | **P** | **Emax (% Nateglinide)** | **SEM** | **P** | **Assay** | **Figure** |
| WT | Nateglinide | - | -4.912 | 0.04627 | - | 93.30 | 2.891 | - | PRESTO-Tango | 3a |
| WT |  | 100 µM (-)-Menthol | -4.969 | 0.06144 | 0.48 | 66.02 | 2.623 | <0.001 |  | 3a |
| WT |  | 300 µM (-)-Menthol | -4.846 | 0.08178 | 0.54 | 56.14 | 3.184 | <0.001 |  | 3a |
| N245S+ T43T | Nateglinide | - | -5.135 | 0.04309 | - | 97.16 | 2.497 | - | PRESTO-Tango | 3b |
| N245S+ T43T |  | 100 µM (-)-Menthol | -5.232 | 0.08857 | 0.34 | 66.04 | 3.317 | <0.001 |  | 3b |
| N245S+ T43T |  | 300 µM (-)-Menthol | -5 | 0.07152 | 0.16 | 54.35 | 2.493 | <0.001 |  | 3b |
| WT | Nateglinide | - | -4.785 | 0.07669 | - | 99.91 | 5.369 | - | PI Hydrolysis | 3c |
| WT |  | 100 µM (-)-Menthol | -5.035 | 0.09378 | 0.0645 | 74.52 | 4.593 | 0.0011 |  | 3c |
| WT |  | 300 µM (-)-Menthol | -5.005 | 0.072 | 0.0594 | 93.48 | 4.347 | 0.3805 |  | 3c |
| N245S+ T43T | Nateglinide | - | -4.744 | 0.2403 | - | 111.3 | 18.43 | - | PI Hydrolysis | 3d |
| N245S+ T43T |  | 100 µM (-)-Menthol | -5.253 | 0.1222 | 0.6998 | 128 | 9.073 | 0.5057 |  | 3d |
| N245S+ T43T |  | 300 µM (-)-Menthol | -5.416 | 0.1554 | 0.4148 | 133.8 | 10.47 | 0.4222 |  | 3d |
