# Supplementary material for: An African-specific haplotype in MRGPRX4 is associated with menthol cigarette smoking
Source: PLoS Genet. 2019 Feb 15;15(2):e1007916. doi: 10.1371/journal.pgen.1007916 (PMC6377114; doi:10.1371/journal.pgen.1007916)
Supplement: S5 Table — (DOCX) [file pgen.1007916.s009.docx]

Table S5. Primers used for genotyping MRGPRX4 SNPs

| **Amplicon** | **Oligo ID** | **Sequence (5’-3’)** | **SNPs genotyped** | |
| --- | --- | --- | --- | --- |
| Amplicon 1 | 1.1F-pcr+seq | AGACTTGCTCTTCGTGTTGTGAAC | | rs2078066; rs16935117  rs7107957; rs2014694  rs1968732; rs1531105;  rs2445182 |
|  | 1.2R-pcr+seq | AGAGGCGTCCATGAGGGGAAATAGGCGATG | |  |
|  | 1.3F-seq | GCTCATGATGGATGCATTCAC | |  |
|  | 1.4R-seq | CAAAATAGAGGTGAGTGAATGC | |  |
|  | 1.5R-seq | CATGCAGATTTCAACTGGAG | |  |
| Amplicon 2 | 2.1F-pcr+seq | CATCGCCTATTTCCCCTCATGGACGCCTCT | | rs1968730; rs2403247  rs1531104; rs11024529 |
|  | 2.2R-pcr+seq | GGCCAGGGCTTTGGACCCGATGATTAA | |  |
|  | 2.3F-seq | CATTGTGTATACCCAGCAG | |  |
|  | 2.4R-seq | GTACACAGTGTTTGGGTAGG | |  |
|  | 2.5R-seq | GAATGCATGCATGAGACTAATGGTAC | |  |
| Amplicon 3 | 3.1F-pcr+seq | ATCTGCGAGCCTCTGTTTCTCTCTT | | rs2468774; rs2445180  rs2445179; rs11024532 |
|  | 3.2R-pcr+seq | TAGAGAGGACAGGGACATGCAAACCAGATA | |  |
|  | 3.3F-seq | GAGTATGCTGAGCGCCATC | |  |
|  | 3.4R-seq | GATGGCGCTCAGCATACTC | |  |
| Amplicon 4 | 4.1F-pcr+seq | TGTCCACACATGGTCACACGTTGGCT | | rs10832895; rs7929457  rs10832896 |
|  | 4.2R-pcr+seq | CACTGTCCCTTTTAAGAACTTTG | |  |
|  | 4.3R-seq | CCAACTGTAAGTTATTATCCGG | |  |
| Amplicon 5 | 5.1F-pcr+seq | TGTTGGTTCTCACCTGGGAC | | rs12791462 |
|  | 5.2R-pcr+seq | AACCTGTGATTTTCAGGTGG | |  |
| Amplicon 6 | 6.1F-pcr+seq | GCTGCTCATGCAGGACAATCTCAGCAACG | | rs2468772 |
|  | 6.2R-pcr+seq | TCAGGTGTTCTTCGCATCTAGCACTTGC | |  |

Note: **1.** pcr+seq = primers used for PCR reaction and Sanger sequencing; **2.** seq = additional primers for Sanger sequencing.
