# Supplementary material for: An African-specific haplotype in MRGPRX4 is associated with menthol cigarette smoking
Source: PLoS Genet. 2019 Feb 15;15(2):e1007916. doi: 10.1371/journal.pgen.1007916 (PMC6377114; doi:10.1371/journal.pgen.1007916)
Supplement: S6 Table — (DOCX) [file pgen.1007916.s010.docx]

| **Table S6. Genotyping results** | | | | | | | | | |
| --- | --- | --- | --- | --- | --- | --- | --- | --- | --- |
|  |  |  |  |  | **Allele frequency** | | | | **P(HWE)** |
| **Chr** | **Position** | **SNP** | **REF** | **ALT** | **Schroeder** | **1KG ASW** | **gnomAD AFR** | **gnomAD EUR** | **Schroeder** |
| 11 | 18192836 | rs2078066 | T | A | 0.2405 | 0.303 | 0.2432 | 0.2786 | 3.05E-05 |
| 11 | 18193020 | rs2014694 | C | G | 0.2385 | 0.303 | 0.2434 | 0.2777 | 1.83E-06 |
| 11 | 18193092 | rs16935117 | G | A | 0.0970 | 0.057 | 0.0759 | 0.0006 | 0.17 |
| 11 | 18193107 | rs7107957 | C | T | 0.1106 | 0.074 | 0.0888 | 0.3344 | 0.32 |
| 11 | 18193305 | rs1531105 | C | T | 0.2381 | 0.303 | 0.2434 | 0.2785 | 2.51E-05 |
| 11 | 18193469 | rs1968732 | T | C | 0.1503 | 0.238 | 0.1644 | 0.2784 | 0.028 |
| 11 | 18193562 | rs2445182 | T | A | 0.2338 | 0.303 | 0.245 | 0.2784 | 2.04E-05 |
| 11 | 18193826 | rs1968730 | A | C | 0.3126 | 0.361 | 0.3357 | 0.2789 | 4.46E-05 |
| 11 | 18193840 | rs2403247 | G | C | 0.0882 | 0.131 | 0.0944 | 0.2971 | 1 |
| 11 | 18194083 | rs1531104 | C | A | 0.2417 | 0.303 | 0.2436 | 0.2787 | 2.03E-05 |
| 11 | 18194348 | rs11024529 | G | T | 0.0094 | 0 | 0.0072 | 0.04364 | 1 |
| 11 | 18194827 | rs2468774 | C | G | 0.2453 | 0.303 | 0.2441 | 0.2864 | 6.47E-05 |
| 11 | 18194878 | rs2445180 | T | G | 0.2402 | 0.303 | 0.2437 | 0.2864 | 3.07E-05 |
| 11 | 18195051 | rs2445179 | C | T | 0.1088 | 0.098 | 0.1008 | 0.0005 | 0.84 |
| 11 | 18195348 | rs11024532 | C | T | 0.0970 | 0.066 | 0.08196 | 0.2886 | 0.17 |
| 11 | 18196571 | rs10832895 | T | G | 0.1046 | 0.066 | 0.0810 | 0.2928 | 0.20 |
| 11 | 18196596 | rs7929457 | A | G | 0.0959 | 0.074 | 0.1216 | 0.0009 | 1 |
| 11 | 18196840 | rs10832896 | T | A | 0.3850 | 0.377 | 0.3915 | 0.318 | 0.15 |
| 11 | 18197936 | rs12791462 | T | C | 0.1047 | 0.139 | 0.1071 | 0.3165 | 0.67 |
| 11 | 18199679 | rs2468772 | A | C | 0.2406 | 0.303 | 0.2493 | 0.2769 | 7.46E-06 |
