# Supplementary material for: An African-specific haplotype in MRGPRX4 is associated with menthol cigarette smoking
Source: PLoS Genet. 2019 Feb 15;15(2):e1007916. doi: 10.1371/journal.pgen.1007916 (PMC6377114; doi:10.1371/journal.pgen.1007916)
Supplement: S7 Table — Part 1. Comparison of WT versus variant values. Part 2. Comparison of WT versus variant menthol response. (DOCX) [file pgen.1007916.s011.docx]

| **Table S7. Association with menthol smoking** | | | | | | | | | | |
| --- | --- | --- | --- | --- | --- | --- | --- | --- | --- | --- |
|  |  |  |  |  |  | **Single SNP association analysis*** | | **R^2^ with** | **Conditional analysis for rs7102322**** | |
| **Chr** | **Position** | **SNP** | **REF** | **ALT** | **N** | **OR (95% CI)** | **P** | **rs7102322** | **OR (95% CI)** | **P** |
| 11 | 18192836 | rs2078066 | T | A | 632 | 1.01 (0.76-1.35) | 0.931 | 0.070 | 7.15 (3.14-16.27) | 2.77E-06 |
| 11 | 18193020 | rs2014694 | C | G | 633 | 0.98 (0.73-1.31) | 0.887 | 0.063 | 7.24 (3.18-16.47) | 2.33E-06 |
| 11 | 18193092 | rs16935117 | G | A | 634 | 1.37 (0.93-2.02) | 0.107 | 0.002 | 6.73 (3-15.13) | 3.86E-06 |
| 11 | 18193107 | rs7107957 | C | T | 633 | 0.84 (0.59-1.19) | 0.330 | 0.001 | 6.48 (2.89-14.53) | 5.77E-06 |
| 11 | 18193305 | rs1531105 | C | T | 632 | 1.01 (0.76-1.35) | 0.940 | 0.067 | 7.12 (3.13-16.2) | 2.85E-06 |
| 11 | 18193469 | rs1968732 | T | C | 632 | 0.99 (0.71-1.38) | 0.946 | 0.014 | 6.57 (2.92-14.76) | 5.19E-06 |
| 11 | 18193562 | rs2445182 | T | A | 631 | 1.01 (0.75-1.35) | 0.965 | 0.056 | 7.02 (3.1-15.93) | 3.09E-06 |
| 11 | 18193826 | rs1968730 | A | C | 635 | 0.95 (0.73-1.25) | 0.729 | 0.051 | 7.31 (3.23-16.57) | 1.89E-06 |
| 11 | 18193840 | rs2403247 | G | C | 635 | 0.79 (0.53-1.17) | 0.237 | 0.005 | 6.41 (2.86-14.37) | 6.62E-06 |
| 11 | 18194083 | rs1531104 | C | A | 635 | 0.98 (0.73-1.31) | 0.886 | 0.064 | 7.25 (3.19-16.5) | 2.28E-06 |
| 11 | 18194348 | rs11024529 | G | T | 635 | 0.77 (0.24-2.43) | 0.653 | 0.005 | 6.64 (2.95-14.92) | 4.69E-06 |
| 11 | 18194827 | rs2468774 | C | G | 636 | 0.98 (0.74-1.31) | 0.896 | 0.067 | 7.37 (3.24-16.75) | 1.87E-06 |
| 11 | 18194878 | rs2445180 | T | G | 635 | 1 (0.75-1.33) | 0.998 | 0.070 | 7.23 (3.18-16.46) | 2.41E-06 |
| 11 | 18195051 | rs2445179 | C | T | 634 | 0.93 (0.64-1.33) | 0.677 | 0.003 | 6.46 (2.88-14.48) | 5.99E-06 |
| 11 | 18195348 | rs11024532 | C | T | 634 | 0.79 (0.55-1.14) | 0.209 | 0.001 | 6.55 (2.92-14.69) | 4.99E-06 |
| 11 | 18196571 | rs10832895 | T | G | 636 | 0.79 (0.55-1.13) | 0.195 | 0.000 | 6.6 (2.94-14.79) | 4.67E-06 |
| 11 | 18196596 | rs7929457 | A | G | 636 | 1.01 (0.68-1.48) | 0.973 | 0.003 | 6.6 (2.94-14.78) | 4.57E-06 |
| 11 | 18196840 | rs10832896 | T | A | 635 | 1.18 (0.94-1.48) | 0.145 | 0.005 | 6.85 (3.05-15.42) | 3.30E-06 |
| 11 | 18197936 | rs12791462 | T | C | 635 | 0.81 (0.56-1.17) | 0.258 | 0.003 | 6.47 (2.89-14.5) | 5.72E-06 |
| 11 | 18199679 | rs2468772 | A | C | 636 | 0.95 (0.71-1.27) | 0.718 | 0.057 | 7.37 (3.25-16.73) | 1.76E-06 |
| * Association with menthol smoking after adjusting for age and gender | | | | | | | | | | |
| ** Association results for rs7102322 after conditioning on the genotype for the listed SNP | | | | | | | | | | |
