# Supplementary material for: An African-specific haplotype in MRGPRX4 is associated with menthol cigarette smoking
Source: PLoS Genet. 2019 Feb 15;15(2):e1007916. doi: 10.1371/journal.pgen.1007916 (PMC6377114; doi:10.1371/journal.pgen.1007916)
Supplement: S8 Table — (DOCX) [file pgen.1007916.s012.docx]

| **Table S8.** | PCR and Sequencing Primers (5'-3') used for RT-PCR of *MRGPRX4* | | | |
| --- | --- | --- | --- | --- |
| Amplicon ID |  |  |  |  |
|  | Oligo ID | Sequence | | |
|  |  |  |  |  |
| Amplicon 1 | 1.1F | GAGTATGCTGAGCGCCATC | | |
|  | 1.2R | GAGGACAGGGACATGCAAAC | | |
|  |  |  |  |  |
| Amplicon 2 | 2.1F | CTGCTGTTTAGTATGCTGGAG | | |
|  | 2.2R | GTAAATTAGGGCCCCCAGAA | | |
|  |  |  |  |  |
| Amplicon 3 | 3.1F | CTGCTGTTTAGTATGCTGGAG | | |
|  | 3.2R | GAGGACAGGGACATGCAAAC | | |
|  |  |  |  |  |
| Amplicon 4 | 4.1F | CAGTCTTCGGTACAAAACTGAC | | |
|  | 4.2R | GATGGCGCTCAGCATACTC | | |
|  |  |  |  |  |
| Amplicon 5 | 5.1F | CAGTCTTCGGTACAAAACTGAC | | |
|  | 5.2R | GATCCACAGAGGATCCTGAC | | |
|  |  |  |  |  |
| Note: F = forward ; R = reverse | | |  |  |
